# Remembering Archbishop Emeritus Desmond Tutu, Global Health Equity and Human Right

**DOI:** 10.11604/pamj.2022.41.14.33062

**Published:** 2022-01-06

**Authors:** Abiola Victor Adepoju, Oluwatoyin Elizabeth Adepoju, Olusola Adedeji Adejumo

**Affiliations:** 1 Jhpiego (an affiliate of John Hopkins University), Federal Capital Territory, Abuja, Nigeria,; 2 Adolescent Friendly Research Initiative and Care (ADOLFRIC), Lagos, Nigeria,; 3 Lagos State Ministry of Health, Lagos, Nigeria

**Keywords:** Archbishop Desmond Tutu, tuberculosis, people living with HIV/AIDS, LGBTQ, global health

## Obituary

It was a rude shock when the demise of Archbishop Emeritus Desmond Tutu was announced to the world on Sunday, the 26^th^ of December, 2021 at 90 years old, after several years of battling with prostate cancer [1]. The Arch, as he was fondly called, will be remembered by different people, nations and groups for different things, but at the core was his lifetime commitment to human right, justice and equality using non-violent approaches. Many would remember him for his campaign against homophobia and child marriage, gender equality and promotion of gay priest, support for the ordination of women, lesbian, gay, bisexual, transgender and Queer (LGBTQ) community, his blessing of same sex union, peaceful activism against apartheid, chairing of the South Africa´s Truth and Reconciliation Commission, support for global health equity and empathy for people living with HIV/AIDS (PLHIV) and tuberculosis [2]. On the international stage, the Arch played a pivotal role in the Israeli-Palestine war; his plea for the release of Nigerian politician, MKO Abiola; stimulation of 'second wave' of democracy in many African countries since 1989; his mediation in the 2007 post-election violence in Kenya. Desmond Tutu was the first black South African to be named Dean of St. Mary's Cathedral in Johannesburg and won the Nobel Peace Prize in 1984 [1,2].

**His childhood woes and health calamities challenged him to care for fellow humans:** Tutu suffered polio which left his right hand permanently weak. At the age of fourteen, he also suffered from tuberculosis (TB) at a time when TB was of epidemic proportions in major world cities like Johannesburg [2]. Tutu coughed out blood and was admitted for a period close to two years at Rietfontein, a sanatorium near Alexandra, leaving him with adhesion in his lungs. During his admission in the hospital, he recounted how he learned about humanity and caring having been inspired by the weekly visits to him (a “ghetto urchin”) by the priest, a man of iconic status with busy daily schedules [2]. He was also lucky to have a bed space for black South African secured for him by one of the anti-apartheid activists, to whom Tutu said he owed his life. His encounter with childhood TB inspired him to study Medicine to which he was admitted but could not proceed to due to lack of financial support from his family. Tutu once noted that 'TB is the child poverty-and also its parent and provider'. Having been stigmatized himself when he suffered TB at the age of 14 and knowing fully well that PLHIV and TB patients were underdogs and oftentimes isolated, he dedicated his life to speak for them and advocated for their rights. He noted earlier that 'all people are children of God and worthy of care'.

**Personal philanthropy towards global health equity and justice:** the Arch also devoted his personal funds to the operation of the nutrition clinic established by Demond Tutu personal physician, Dr Ingrid Le Roux. Tutu and his wife continued to support the work of Stellenbosch University TB researchers and experts like Prof Nulda Beyers and the mobile health clinics for the South African poorest communities through the 'Good Clean Health' Train initiative [3].

**Campaign against global health injustice using the power of the pen, pulpit and popularity:** Archbishop Tutu was a global voice and unflinching campaigner against social injustice. He was at the forefront of the fight against discrimination of PLHIV and particularly LGBT community, women, children and advocated for treatment access for all irrespective of gender, race, ethnicity, sexual orientation and income [2]. He was also outspoken against the patency law stating that 'people and not profits, must be at the center of global pharmaceutical patency law'. As a community influencer and role model, Archbishop Tutu influenced large-scale community participation in the mobile health initiatives such as: 'Tutu Testers', 'Tutu Trucks' among others. Archbishop Tutu's opening speech at the AIDS 2016 conference in Durban, South Africa clearly reflected his philosophy which aligned with the conference theme: “Access equity rights now,” a call to leave no one behind and provide comprehensive HIV services to everyone in need, overcoming the marginalization of vulnerable populations, challenging discriminatory laws and championing a community-centred and rights-based response to HIV. As a prolific writer, following the Anglican Church´s anti-LGBTQ declaration in 1998, Tutu wrote the former archbishop of Canterbury, George Carey, vehemently underscoring his disappointment and shame of belonging to the Anglican church [2]. He was quoted as saying several times that he would neither go to a homophobic heaven nor worship a God who is homophobic [1]. On March 12, 2010, the Arch wrote an editorial in the Washington Post titled: “In Africa, a step backward on human rights”. The article highlighted how African gays and lesbians lived in fear and hiding without any form of protection from state apparatus, aside stigma and punishment [4]. Tutu further condemned discrimination of LGBTQ community in accessing life saving antiretroviral therapy and compared such action with the 'evil of apartheid' or 'a new apartheid' He further observed that 'hate has no place in the house of God'.

**His commitment to global health research that improved population health:** Tutu has been described as someone who was always inquisitive about recent research progress and innovations in the field of TB, HIV and related disease. He celebrated scientific successes, exploits and losses as well. He customarily appreciated researchers through congratulatory messages and emissaries. Several health-focused research centers and foundations were named after Desmond Tutu among which are: Desmond Tutu Center for TB Research and Desmond Tutu Center for HIV Research both affiliated with Desmond Tutu Health Foundation [3]. Tutu was excited to have these centers named after him in 2004. The centers have focused on research in the areas of TB/HIV Prevention, TB and HIV vaccine candidates, pediatric research and child drug formulations, operational research, clinical trials, treatment delivery and adherence as well as socio-behavioral research. Awards won by the Desmond Tutu centers for TB and HIV research include the Royal Society Pfizer Award given to Prof Linda-Gail Bekker for her work on TB/HIV collaboration. During the 2012 Stop TB Symposium, an annual event at the World Conference of the International Union Against Tuberculosis and Lung Disease, the Desmond Tutu TB Center at Stellenbosch University was awarded the Stop TB Partnership Kochon Prize for its ground-breaking research on childhood TB and for pioneering community-based approaches to TB and HIV care, improving the health of vulnerable groups, with a special emphasis on children and families [5]. The centers have also been trademarks for ground breaking national and international researches such as the Truvada HIV prevention trial; the PETITE trial aimed at expanding ART options for HIV prevention and treatment in neonates; the BENEFIT (Better Evidence for Improved MDR-TB treatment for children) kids project and the SHINE trial which investigated shortening of TB treatment duration for children.

The ideology of a man rooted in simple conviction that every man is created equal before God and deserves the same respect, dignity and justice which he applied whether it was racism, HIV/AIDS discrimination, homophobia, or economic and social inequality. As the world mourn the exit of Archbishop Tutu, we need to emulate his commitment to resilience, compassion and speaking truth to power, the necessary tools the world would need to fight the many pandemics.
